# Autism Spectrum Disorder, Oral Implications, and Oral Microbiota

**DOI:** 10.3390/children12030368

**Published:** 2025-03-15

**Authors:** Emiliana D’Angelo, Fausto Fiori, Giuseppe A. Ferraro, Assunta Tessitore, Luca Nazzaro, Rosario Serpico, Maria Contaldo

**Affiliations:** 1 Multidisciplinary Department of Medical-Surgical and Dental Specialties, University of Campania Luigi Vanvitelli, Via Luigi de Crecchio, 6, 80138 Naples, Italy; emiliana.dangelo@studenti.unicampania.it (E.D.); fausto.fiori@unicampania.it (F.F.); rosario.serpico@unicampania.it (R.S.); 2 Department of Medicine and Health Sciences “Vincenzo Tiberio”, University of Molise, 86100 Campobasso, Italy; giuseppe.ferraro@unimol.it; 3 Department of Clinical Medicine, Public Health, Life Sciences and Environment, University of L’Aquila, Piazzale Salvatore Tommasi 1, Blocco 11, 67010 L’Aquila, Italy; assunta.tessitore@student.univaq.it; 4 Division of General, Oncological, Mini-Invasive and Obesity Surgery, University of Campania “Luigi Vanvitelli”, 80131 Naples, Italy; luca.nazzaro@studenti.unicampania.it

**Keywords:** autism spectrum disorder, dysbiosis, oral microbiota, oral health, oral hygiene, microbial dysbiosis

## Abstract

**Background/Objectives**: Autism spectrum disorder (ASD) is a complex neurodevelopmental disorder characterized by difficulties in social interaction, communication, and repetitive behaviors. Recent evidence indicates a significant relationship between ASD and imbalances in microbiota, particularly in the oral and gastrointestinal areas. This review examines the impact of oral microbiota, self-injurious behaviors (SIB), sensory sensitivity, and dietary choices on the comorbidities associated with ASD. **Methods:** An extensive literature review was conducted using PubMed and Scopus. The focus was on human studies with full-text availability, utilizing search terms related to ASD, oral health, oral microbiota, and neurodevelopmental disorders. The research was evaluated for methodological quality and its relevance to the connections between microbiota, oral health, and ASD. **Results:** Individuals with ASD face unique oral health challenges, including injuries from self-injurious behaviors and increased sensory sensitivity, which complicate oral hygiene and care. Selective eating can lead to nutritional deficiencies and worsen oral health issues. Dysbiosis in oral and gut microbiota, marked by altered levels of acetate, propionate, and butyrate, interferes with gut-brain and oral-brain connections, contributing to behavioral and neurological symptoms. Treatment options such as probiotics, fecal microbiota transfer, and sensory integration therapies can potentially alleviate symptoms and improve quality of life. **Conclusions**: The relationship between ASD, oral health, and microbiota suggests a bidirectional influence through neuroinflammatory mechanisms and metabolic disturbances. Proactive strategies focusing on microbiota and dental health may help reduce comorbidities and enhance the overall management of ASD, underscoring the need for further research into microbiota–host interactions and their therapeutic potential.

## 1. Introduction

### 1.1. Autism Spectrum Disorder (ASD) and Associated Characteristics

**Figure 1:**
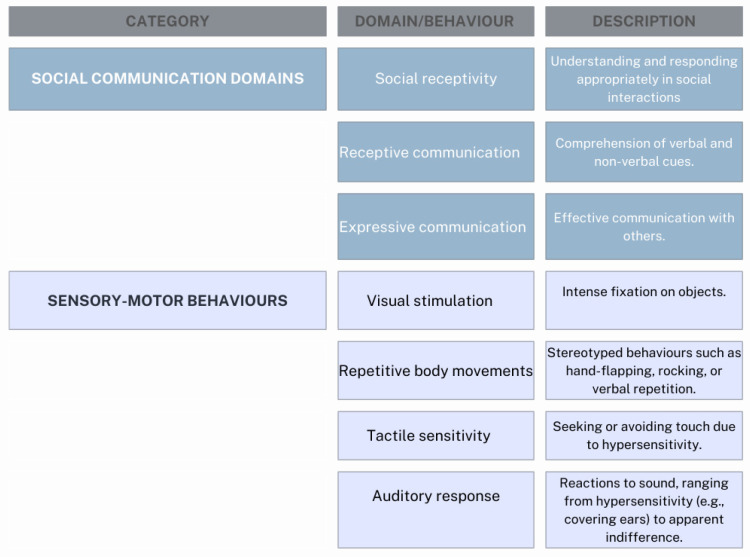
Domains of social communication, adapted from “Masiran et al. [11], and Lord C. et al. [12]”.

Autism spectrum disorder (ASD) refers to a heterogeneous group of lifelong neurodevelopmental disorders characterized by deficits in social communication, social interaction, and sensory-motor behaviors, often accompanied by restricted and repetitive interests or activities [1]. ASD is typically diagnosed in early childhood (around 2–3 years), with many of the most evident symptoms emerging at this stage (Figure 1). Some children may initially develop typically before losing previously acquired skills or halting developmental progress [2,3]. ASD was first identified by American child psychologist Leo Kanner, who described 11 children with distinct behavioral patterns, hypothesizing an innate trait limiting their socialization abilities. Historically termed “early infantile ASD” or “Kanner’s ASD,” the condition is now classified under the broader term Autism Spectrum Disorder (ASD), reflecting the wide variability in symptom severity and presentation [4,5]. The U.S. National Institute of Child Health and Human Development defines ASD as a “complex biological disorder beginning before the age of three that causes delays or challenges in multiple aspects of development” [6]. ASD is now categorized as a neurodevelopmental disorder and encompasses previous subtypes such as Asperger’s syndrome and pervasive developmental disorder not otherwise specified (PDD-NOS). These subtypes are no longer used due to diagnostic inconsistencies [1]. According to the most recent edition of the Diagnostic and Statistical Manual of Mental Disorders (DSM-V-TR) from the American Psychiatric Association, neurodevelopmental disorders are characterized by early-onset impairments in personal, social, academic, or occupational functioning due to atypical brain development [1]. These disorders encompass many conditions that manifest in early childhood and persist throughout life. The DSM-5-TR classifies neurodevelopmental disorders into several categories, including intellectual disabilities, communication disorders, autism spectrum disorder (ASD), attention-deficit/hyperactivity disorder (ADHD), specific learning disorders, and motor disorders such as developmental coordination disorder, stereotypic movement disorder, and tic disorders (e.g., Tourette’ syndrome).

ASD, as defined by the DSM-5-TR, is diagnosed based on persistent deficits in social communication and social interaction across multiple contexts, along with restricted, repetitive patterns of behavior, interests, or activities [1].

Diagnosis relies on behavioral assessment, as no reliable biomarkers currently exist. ASD stems from early brain development anomalies and neural reorganization. While the exact causes remain unclear, evidence highlights a significant genetic component. Families with one child with ASD face a 20-fold higher risk of having another affected child [7]. Environmental factors, such as maternal infections during pregnancy (e.g., rubella or cytomegalovirus), advanced parental age, and exposure to certain medications or toxins, are also implicated [8]. Despite extensive research, specific biological markers and structural or functional brain differences—such as those in the cerebellum, amygdala, hippocampus, frontal cortex, and brainstem nuclei—remain inconclusive. The purported link between vaccinations and ASD has been consistently disproven [9]. Over the past 50 years, ASD has evolved from being considered a rare, childhood-onset disorder to a more common and diverse condition. In Italy, one in 77 children aged between 7 and 9 is estimated to have ASD, with a higher prevalence among boys (4.4 times that of girls) [10]. This increase may be due to improved diagnostic methods, greater awareness, and enhanced medical training, though no universally accepted explanation exists [6].

To meet the diagnostic criteria for ASD, an individual must exhibit persistent difficulties in social communication and interaction across multiple contexts, as well as restricted, repetitive patterns of behavior, interests, or activities, as defined by the *DSM-V-TR* of the American Psychiatric Association [1].

Individuals with ASD often experience comorbid neurological, psychiatric, and medical conditions, such as genetic syndromes (e.g., fragile X syndrome) and attention-deficit/hyperactivity disorder (ADHD). Additionally, due to dietary habits, self-care challenges, self-injurious behaviors, and medication use, they are at heightened risk for chronic oral health conditions [13].

**Figure 2:**
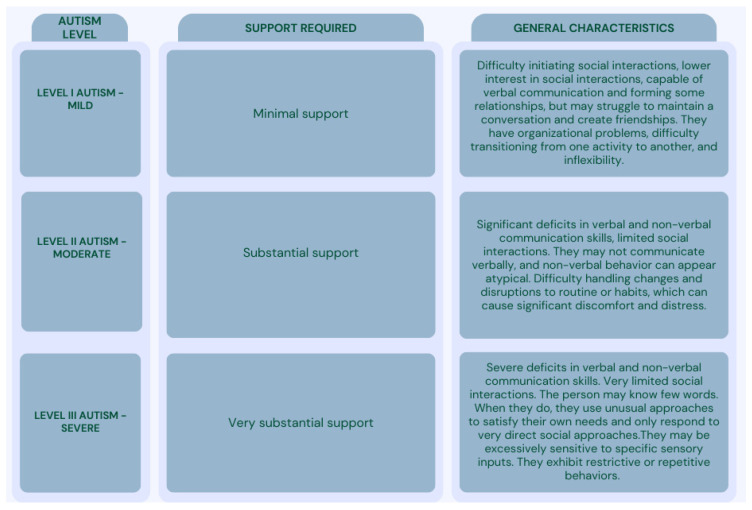
ASD level classification adapted from *DSM-5* [1], Lord C et al. [12], and Sandin S et al. [8].

ASD’s complexity ranges from mild to severe (Figure 2), underscoring the importance of effective early monitoring and intervention to address developmental challenges. Continued research into its multifactorial causes and diverse presentations remains essential for improving outcomes.

### 1.2. Pathogenetic Hypothesis

The pathogenetic hypothesis seeks to clarify the distinctive functioning of the autistic mind by analyzing the cognitive, social, and emotional abilities of individuals with ASD [8,12]. Meanwhile, other research focuses on investigating brain function to uncover potential neurobiological bases of the syndrome. Neuroimaging, neurofunctional studies, and neurotransmitter analysis attempt to connect behavioral symptoms with neurophysiological foundations [14,15].

Despite these studies, it remains impossible to identify consistent morphological and biochemical alterations across the different autism spectrum disorders. However, several pathogenic theories have been proposed and seem to be supported in specific subgroups of individuals with autism spectrum disorder. These include abnormal development of particular brain structures, altered connectivity between various brain regions, neurotransmitter dysfunctions in the central nervous system, immunological anomalies, autoimmune processes, and metabolic disorders, all of which confirm the multifactorial origin of the condition [16,17].

Acknowledging both the biological and hereditary components of ASD, research has shifted towards identifying specific genetic causes. This effort involves searching for genes linked to autism spectrum disorders. Genome studies and cytogenetic analyses conducted on families with multiple affected members have led to the discovery of various ASD-associated genes located on chromosomes 15, 16, and 22 and the X chromosome [18,19,20,21]. However, research in this area is continually evolving, and the involvement of genes on other chromosomes cannot be ruled out. Currently, no single gene has been identified as solely responsible for ASD; instead, the condition has a complex genetic origin involving multiple genes. Some of these genes regulate the production of molecules essential for the development and maintenance of neural networks, including the following:

- Proteins involved in synaptic communication, which enable interactions between nerve cells;
- Factors regulating gene expression to determine which genes are activated;
- Neurotransmitters and receptors, which transmit nerve signals between cells via synapses;
- Genes related to brain development, which influence the growth and organization of the brain.

This genetic complexity reflects the variety of manifestations of ASD and the ongoing efforts to understand its biological foundations. None of the genes identified so far in specific groups of patients or their families can, on their own, account for all the symptoms of ASD, which is a complex neuropsychiatric disorder. For an individual to develop ASD, they must inherit a particular combination of “defective” genes. However, this genetic combination, while a potential cause of the disorder, may not suffice independently. In many cases, ASD may only manifest if environmental epigenetic triggers activate these genes. These triggers are thought to include exposure to infections during prenatal life, disruptions in maternal-fetal immune states, and contact with drugs or toxic substances, including certain foods, during pregnancy [22,23,24,25,26,27]. Consequently, the role of genes is increasingly recognized as a predisposing factor that interacts with environmental elements to determine the onset of the disorder. Therefore, ASD can be understood as the result of a complex interaction between genetics and the environment, with genetics serving as a predisposing foundation rather than a sole cause.

### 1.3. Aims

This study aimed to examine the effects and consequences of ASD on oral health and how microbiome imbalances may influence neurodevelopment through the gut–brain and oral–brain axes. It also explores how ASD-related behaviors and diets affect oral and gut bacteria. Additionally, the study tests microbiome-based treatments, such as probiotics and dietary changes, to improve oral health and behavior for future tailored diagnostic and therapeutic strategies.

## 2. Materials and Methods

The review examined relevant literature using PubMed and Scopus as the primary search databases. The search strategy involved combining keywords and synonyms while applying Boolean operators (AND, OR) to explore topics such as *ASD*, autism spectrum disorder (ASD), oral microbiome, gut microbiome, dysbiosis, the gut–brain axis, neurodevelopmental disorders, oral health, probiotics, food selectivity, genetic-environmental interactions, behavioral symptoms, and microbial diversity. The investigation did not impose any restrictions—the eligibility criteria required original human studies that were available in full text and published in English. Studies using animal or cellular models, those published in languages other than English, and those unavailable in full text were excluded. The research team also reviewed and considered articles, letters, conference proceedings, meeting abstracts, and editorials to identify potentially relevant references for the present overview. The eligibility was assessed by two early career researchers (E.D.A. and A.T.) and a third senior researcher (F.F.), who supervised them in the choice based first on the title/abstract and then on the full-text reading.

## 3. Results

The literature review highlights a complex interaction between ASD, oral health, and microbiota, with a strong correlation between microbial alterations and the clinical manifestations of the disorder. Individuals with ASD have a higher incidence of self-injurious behaviors (SIB), sensory hypersensitivity, and food selectivity, which contribute to an increased risk of oral pathologies, including traumatic lesions, gingivitis, enamel hypoplasia, and oral microbiota imbalances.

Evidence suggests that dysbiosis of the oral and gut microbiota may play a key role in neuroinflammatory processes and behavioral disorders associated with ASD. Alterations in acetate, propionate, and butyrate levels can affect the gut–brain and oral–brain axes, contributing to the clinical manifestations of ASD.

Recent studies indicate that therapeutic approaches aimed at modulating the microbiota, such as probiotics and fecal microbiota transplantation, could improve oral health and certain behavioral aspects of ASD.

However, further research is needed to determine the long-term effectiveness of these strategies.

These findings support the importance of integrated therapeutic strategies, including oral health management, nutritional interventions, and microbiota-targeted treatments, to enhance the quality of life for individuals with ASD.

### 3.1. Self-Injurious Behavior (SIB) and Oral Lesions

Self-injurious behavior (SIB) refers to a range of harmful actions that are self-inflicted without the conscious intent to commit suicide. These behaviors can include biting, hitting, and excessive scratching. For a behavior to be classified as self-injurious, it must meet certain criteria: it is socially unacceptable, repetitive, and causes mild to moderate tissue damage [28,29].

**Figure 3:**
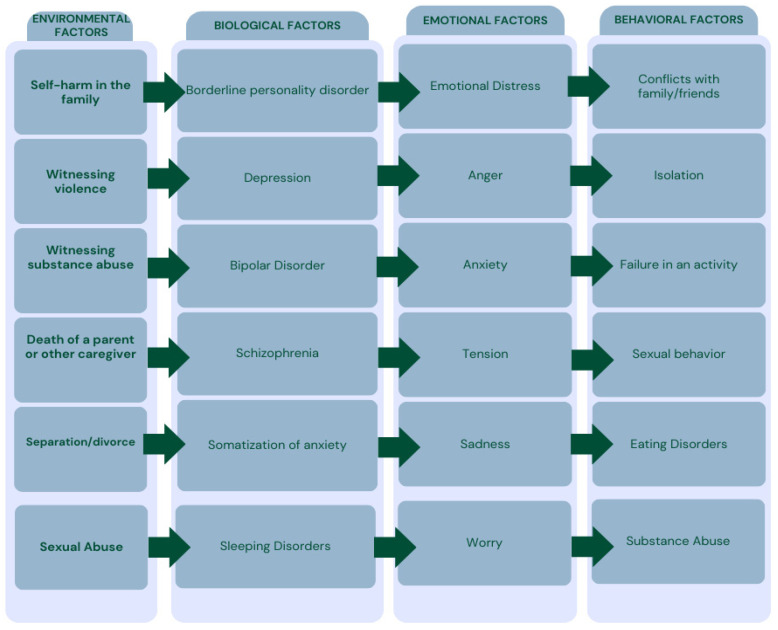
Risk factors associated with self-harm adapted from Simeon et al. [30] and the American Psychiatric Association’s *DSM* [1].

Simeon and Favazza identified a series of environmental, biological, emotional, and behavioral risk factors associated with self-harm (Figure 3) and proposed categorizing self-injurious behaviors into four types as follows [30]:

- Stereotypic, including head-banging, self-hitting, biting, and scratching.
- Major, often associated with psychosis and causing significant harm, such as self-enucleation, self-castration, and self-amputation.
- Compulsive, referring to behaviors like hair-pulling, skin-picking, and nail-biting, often linked to conditions such as trichotillomania, stereotypic movement disorder, or obsessive-compulsive disorders.
- Impulsive, encompassing skin-cutting and burning, associated with borderline personality disorder, antisocial personality disorder, post-traumatic stress disorder (PTSD), and eating disorders.

SIB is often observed in individuals with neurological developmental delays, particularly in children and adults with ASD, although not all individuals with ASD exhibit SIB, and its occurrence can vary widely among different individuals [31]. Approximately 75% of these injuries are localized to the head and neck region, affecting oral structures like the gums, buccal mucosa, tongue, periodontal tissues, and/or teeth [32,33].

The oral lesions resulting from SIB can pose diagnostic challenges, especially when they are the first or only indication of an underlying disease. Common oral findings in patients with ASD often include traumatic ulcerative lesions, usually resulting from head-banging, facial tapping, or gum-picking. Another noted consequence of self-injury in ASD is self-extraction, wherein individuals remove their own teeth [34,35,36]. Unusual oral habits associated with ASD may also include bruxism (teeth grinding), tongue thrusting, chewing non-nutritive objects such as gravel, cigarette butts, or pens, and repeated regurgitation [37,38,39,40].

### 3.2. Consequences of Dermatillomania in the Oral Cavity

Dermatillomania, also known as skin picking, psychogenic excoriation, or neurotic excoriation, is a condition characterized by repetitive picking or pulling at the skin. This behavior can lead to damage to the skin and soft tissues, significant distress, and functional impairment [1,41]. It is classified as a mental disorder in the Diagnostic and Statistical Manual of Mental Disorders (DSM-5) under the category of “obsessive-compulsive and related disorders” (OCRD) [41,42]. Skin picking is often associated with various psychiatric conditions, including ASD, alcohol abuse, mood disorders, obsessive-compulsive disorder (OCD), body dysmorphic disorder, anxiety, and borderline personality disorder. It is frequently underdiagnosed and can occur at any age, although its onset is typically during adolescence, often coinciding with puberty [43].

To be diagnosed with skin picking disorder, the following criteria must be met [43]:

- Recurrent skin picking that results in lesions;
- Repeated attempts to reduce or stop the behavior;
- Significant clinical distress or impairment in social, occupational, or other important areas of functioning caused by the behavior;
- The skin picking is not attributable to the physiological effects of a substance or another medical condition;
- The behavior is not better explained by the symptoms of another mental disorder.

The severity of dermatillomania can range from mild to severe. The behavior often begins with the individual inspecting their skin or feeling irregularities in the skin’s surface. Some individuals may pick at completely healthy areas of skin [43]. Skin-picking behaviors can be categorized into two forms: automatic picking, which occurs almost unconsciously during routine activities, and targeted picking, which is more deliberate and may be driven by the belief that scabs, lesions, or blemishes need to be removed, or by physical sensations such as itchiness or tingling [43].

Factors contributing to this disorder include difficulties in emotion regulation, poor impulse control, heightened skin sensitivity, and a low tolerance for perceived skin imperfections. In ASD subjects, targeted picking may act as a coping mechanism to alleviate internal discomfort, while automatic picking often serves as a form of self-stimulation. Many individuals report experiencing immediate relief during the act of picking, which is frequently followed by feelings of frustration or anger.

Dermatillomania can lead to various consequences for the skin and oral health due to the compulsive behaviors of scratching, picking, or pulling. These may include the following:

- Skin lesions, such as abrasions and ulcerations on the lips, mouth, or fingers;
- Infections, as damaged skin becomes more vulnerable to bacterial or fungal infections;
- Scarring, particularly in areas where the skin is thin, like the lips, potentially leading to permanent marks;
- Pain and discomfort, which can affect the individual’s quality of life;
- Dental complications, if oral tissues are involved, such as irritation or cuts from scratching or picking;
- Oral health issues, possibly stemming from frequent contact with teeth or oral structures during the behavior.

It results in a significant impact on quality of life, with psychological and social repercussions resulting from the physical and emotional effects of the condition.

### 3.3. Food Selectivity in Patients with ASD and Impacts on the Oral Cavity

The term “food selectivity” refers to food refusal, a limited variety of food intake, and, in severe cases, consuming only a single food or liquid [44]. These behaviors are influenced by medical, sensory, and behavioral factors, as well as heightened sensory sensitivities and restricted interests [45].

In general, during meals, young children explore food using their senses, contributing to their self-awareness through the perceptions of taste, touch, and smell. These experiences, which involve imitation and reciprocal exchange, are shaped by an environment that provides support, affection, and enjoyment. Family eating habits, including food preferences, play a significant role in children’s eating behaviors. They can facilitate the development of functional feeding or contribute to issues such as being overweight, junk food consumption, and other eating disorders by promoting unhealthy eating habits [46].

In the latest edition of the *DSM-5*, the classification of feeding and eating disorders during childhood includes pica, avoidant/restrictive food intake disorder (ARFID), rumination disorder, and eating disorders [47]. The term “pica” derives from the Latin word pīca, meaning “magpie,” as the bird is known for collecting and ingesting various unusual non-nutritional objects. This condition involves the persistent consumption of non-nutritive substances and is typically diagnosed when the behavior persists for at least one month. Pica can be linked to conditions such as iron deficiency, developmental disorders, or psychiatric conditions [48].

Avoidant/restrictive food intake disorder (ARFID) is characterized by avoiding or restricting food intake, leading to reduced calorie and nutrient absorption. Rumination disorders occur when undigested or partially digested food is repeatedly regurgitated from the stomach. Eating disorders reduce food consumption and impact the subject’s physical health and psychosocial functioning [49].

Feeding issues in ASD children may be associated with the persistent consumption of non-nutritive substances, atypical use of utensils, specific food preparation preferences, and predilection based on food texture (such as soft, gelatinous, crunchy, or hard), taste, smell, color, shape, or temperature. Environmental sensory stimuli during mealtimes also contribute to these behaviors [50]. Behavioral expressions may include using the same utensils, insisting on consistent food presentation (e.g., food must always be prepared the same way), accepting only specific brands, and showing attention to packaging. Children with ASD refuse more food than neurotypical peers, primarily accepting low-consistency foods like purées [51]. They also consume significantly less fruit, dairy, vegetables, proteins, and starches [52] and are more reluctant to try new foods, exhibiting food neophobia [53].

Food selectivity often persists over the years, highlighting the need for early interventions in childhood to increase variety and promote healthy eating habits.

Children with ASD have lower protein, calcium, phosphorus, selenium, vitamin B12, and vitamin D intake while consuming higher levels of magnesium, polyunsaturated fatty acids, and vitamin E compared to their neurotypical peers [54]. Certain age groups may consume excessive amounts of sodium, folate, manganese, zinc, vitamin A (retinol), selenium, and copper [55]. Children with ASD aged 2 to 5 are more likely to be overweight or obese, whereas those aged 5 to 11 are more prone to being underweight. [56,57]. They are also at a higher risk of developing gastrointestinal disorders, particularly among females, which are linked to more severe food selectivity [58].

Healthcare providers must assess whether feeding problems are organic or behavioral. For example, symptoms such as vomiting could be associated with biomedical factors like gastrointestinal reflux and significant nutritional deficits [59]. Additionally, food allergies are more prevalent among individuals with ASD. It is essential to recognize that medical and behavioral issues may coexist during the same developmental stage [60].

Furthermore, there is a strong correlation between ASD, feeding disorders, and gastrointestinal microbiota alterations, which can potentially lead to gastrointestinal issues such as constipation, vomiting, diarrhea, and abdominal pain. Indeed, restricting food groups that modulate the gut microbiota, such as fruits, vegetables, and fibers from certain grains, may trigger intestinal dysbiosis, thus causing the increased prevalence of *Enterobacteriaceae*, *Salmonella*, *Escherichia/Shigella*, and *Clostridium* XIVa, accompanied by an aberrant immune response and leaky gut [61,62].

Food selectivity may stem from sensory processing difficulties and can be viewed as an expression of altered sensory responses and behavioral rigidity. According to the literature, more children with ASD show atypical sensory processing than those without ASD [63]. Among children with ASD, those with atypical oral sensory sensitivity are more likely to avoid a broader range of foods and consume fewer vegetables than those with typical oral sensory sensitivity [64].

Sensory sensitivity, also known as sensory defensiveness or sensory over-reactivity, was first described by Ayres in some children with ASD. It is an exaggerated reaction to touch, often followed by negative behavioral responses, which would not provoke any reaction in most neurotypical individuals [65].

Children with tactile defensiveness even struggle with being hugged and avoid physical contact. Some researchers suggest that atypical sensory processing could be one of many diagnostic criteria for ASD. Oral defensiveness, considered a component of tactile defensiveness, is the avoidance of certain food textures and mouth-related activities, such as brushing teeth. Excessive oral reactivity inevitably leads to feeding difficulties and food selectivity [66].

In cases of oral under-reactivity, children may not perceive sensations correctly, triggering behaviors like putting objects in their mouths to stimulate them.

Children with tactile defensiveness exhibit significant differences in eating habits, hesitate to eat unfamiliar foods, and avoid eating at other people’s homes due to preparation procedures, odors, and temperatures [67]. Research shows that children with tactile defensiveness may engage in self-injurious behaviors such as lip-biting and cheek-biting. Furthermore, children with ASD struggle to tolerate certain tactile materials, such as wool and clothing against their skin, highlighting that hypersensitivity affects all sensory organs.

Environmental factors (family eating habits) play a role in developing and maintaining food refusal. Parents of children with food selectivity experience higher stress levels than parents of children without these issues [68]. Additionally, limited food intake in children significantly impacts family habits, as children exhibit irritability, tantrums, reluctance to sit at the table, or behaviors like throwing or spitting food, disrupting typical mealtime routines [69]. To avoid such behaviors, parents often cater to the child’s preferences, excluding certain foods to the detriment of other family members [70].

When designing clinical interventions, healthcare providers must consider the need to support families through appropriate parent training sessions.

#### 3.3.1. Oral Consequences of Food Selectivity

Food selectivity often leads to nutritional deficiencies, and serious health consequences have been reported in children and young individuals with restrictive diets. These include jaundice, anemia, scurvy, rickets, gingivitis, and hypogonadism. Nutrients are fundamental to human metabolism, supporting growth, recovery, and disease defense.

**Figure 4:**
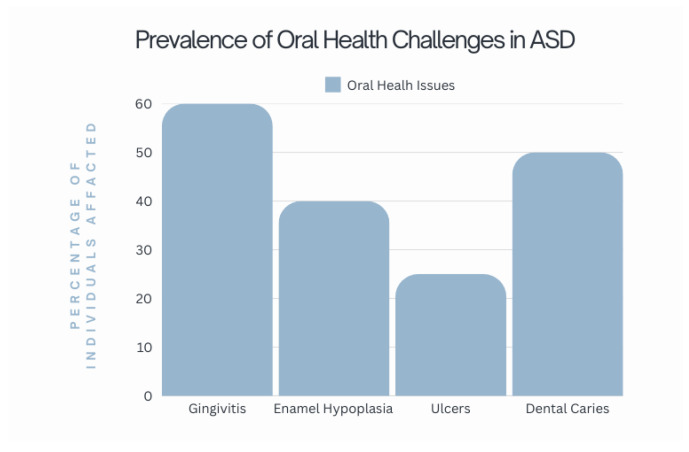
Prevalence of oral health challenges in ASD adapted from Adams et al. [72], Cermak et al. [51], and Blanchard et al. [73].

**Figure 5:**
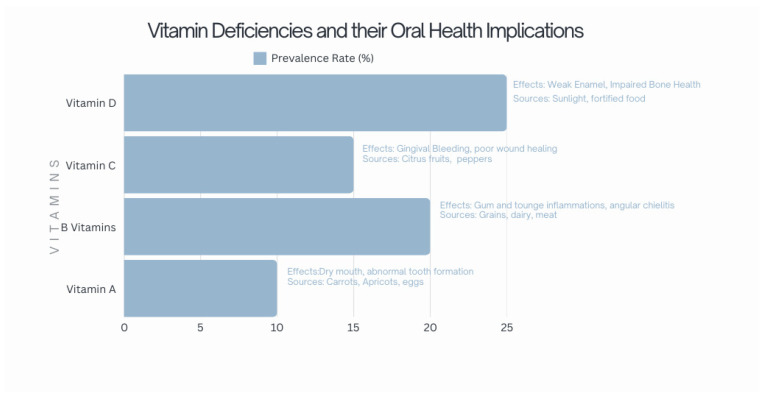
Vitamin deficiencies and their oral health implication adapted from Gandhi et al. [88], Firth et al. [82], and Aponte et al. [52].

Nutritional imbalances caused by food selectivity can significantly affect oral health, potentially leading to issues like gingivitis, delayed wound healing, and increased susceptibility to infections in the oral cavity [71] (Figure 4 and Figure 5).

##### Vitamin A Deficiency

Vitamin A is found in plant-based foods (e.g., carrots and apricots) and animal-derived products (e.g., eggs and certain meats) [74]. Its deficiency in the oral cavity is linked to reduced epithelial tissue development, oral mucosa hyperkeratosis, a sensation of roughness, dry mouth, and abnormal tooth formation [75]. Furthermore, a lack of vitamin A can negatively impact periodontal health and is associated with enamel hypoplasia, a condition characterized by insufficient enamel on the tooth’s surface. Enamel hypoplasia, caused by disruptions during enamel secretion, calcification, or maturation, increases tooth sensitivity and susceptibility to damage, including a heightened risk of dental caries [76,77].

##### B Vitamins Deficiencies

Deficiencies in B vitamins manifest in various oral symptoms, such as aphthous ulcers, chapped lips, and inflammation of the tongue and gums [78,79]. Specific deficiencies in vitamins B6 and B12 may result in angular cheilitis, exfoliative cheilitis, hypertrophic glossitis, and atrophic glossitis [80].

##### Vitamin C Deficiency

Scurvy, caused by vitamin C deficiency, is marked by gingival bleeding, arthralgia, skin discoloration, delayed wound healing, perifollicular hemorrhage, and bruising. While its prevalence has declined, scurvy still occurs in individuals with unbalanced diets [81]. Gastrointestinal bleeding and other symptoms may also manifest. Vitamin C deficiency impairs collagen synthesis, weakening vascular walls and causing metabolic disturbances. Fortunately, scurvy responds well to high doses of oral ascorbic acid, though relapses can occur. Healthcare professionals must recognize its symptoms, especially in societies with uncommon nutritional deficiencies [82,83].

##### Vitamin D Deficiency

Vitamin D is vital for bone formation and remodeling. Severe deficiencies in children can result in imperfect amelogenesis and dentinogenesis and negatively impact periodontal health [84,85]. Conversely, some authors have identified a potential association between ASD susceptibility and severity and some genetic variations in the vitamin D pathway and absorption [86,87].

### 3.4. Microbiota and Microbial Diversity in Patients with Autism Spectrum Disorders

The microbiota is the community of trillions of commensals, symbiotic, and pathogenic microorganisms inhabiting human and animal bodies [89]. External factors, including the environment, diet, and lifestyle, influence the vitality of the microbiome [90]. The microbiome composition varies significantly among individuals and is influenced by diet, lifestyle, stress, socioeconomic conditions, work environment, and diseases [91].

The gastrointestinal (GI) microbiome plays a crucial role in mammalian physiology, supporting essential processes such as digestion, synthesis, and absorption of vital nutrients, including amino acids, folates, and B vitamins [92]. Increasing evidence suggests that the GI microbiome can also impact host behavior and neurodevelopment through a mechanism known as the “gut-brain-microbiome axis” [93,94]. This axis represents a bidirectional communication pathway mediated by microorganisms between the central nervous system (CNS) and the digestive tract. The communication occurs through various channels, including direct neural activation, immune system modulation, hormonal, peptide, and epigenetic signaling [95].

Although the precise mechanisms remain unclear, alterations in the GI microbiome and the gut–brain axis have been identified in various neuropsychiatric and neurodevelopmental disorders, including autism spectrum disorder (ASD) [96]. Indeed, many individuals with ASD exhibit gastrointestinal comorbidities, such as constipation, chronic diarrhea, abdominal pain, and gastroesophageal reflux [97]. While nearly 50% of the risk of developing ASD is attributed to genetic factors, such as single-nucleotide polymorphisms and copy number variants [8], it is plausible that gene–environment interactions, influenced by the GI microbiome, may play a significant role in modulating ASD risk [98].

The microbiome’s influence on serotonin levels offers a compelling example of how these microorganisms can affect neurobiological processes [99]. Polymorphisms in the serotonin transporter gene are known to increase the risk of ASD [100]. However, most serotonin synthesis occurs in enterochromaffin cells in the gut, where tryptophan is converted to serotonin via the enzyme tryptophan hydroxylase [101]. This process is enhanced and stimulated by the GI microbiome, mainly through the effects of short-chain fatty acids [102]. Once synthesized, serotonin acts locally in the gut to promote intestinal motility. A small portion may enter peripheral circulation and influence the CNS, especially during early brain development [103]. Therefore, disruptions in the gut microbiome could affect serotonin signaling, interacting with a child’s genetic predisposition and contributing to conditions like ASD [89].

Evidence of disrupted gut–brain interactions in ASD is becoming increasingly robust [104]. For example, a study involving 13 children with regressive ASD and gastrointestinal comorbidities reported elevated levels of fecal *Clostridium* and non-sporulating anaerobic bacteria compared to typically developing (TD) children [98]. Other studies have confirmed altered *Clostridium* abundance in the feces of individuals with ASD [105]. Variations in the *Bacteroides*-to-*Firmicutes* ratio have also been observed in children with ASD, although findings regarding the direction of these changes remain inconsistent [106,107,108]. Additional research has highlighted alterations in the presence of *Lactobacillus*, *Prevotella*, *Sutterella*, *Desulfovibrio*, and the *Veillonellaceae* family in ASD populations [72,109].

Discrepancies among studies may stem from various factors, including small sample sizes, making it challenging to draw definitive conclusions about such a heterogeneous condition as ASD. These limitations have also hindered the ability to categorize individuals with ASD into distinct phenotypic subtypes. Larger-scale research could provide valuable insights into the relationship between the gut microbiome, autistic behavior, gastrointestinal conditions, and immune functionality, enabling a deeper understanding of the complex interactions underlying ASD development.

#### 3.4.1. A Hypothesized Correlation Between Microbiota and Autism Spectrum Disorder

The role of the gut–brain axis in mental health disorders has been the focus of numerous studies. Recently, attention has shifted to oral microbiota, given that the mouth represents the starting point of the digestive tract. Oral microbiota may influence mental disorders by entering the bloodstream, triggering inflammatory responses, or reaching the brain through the trigeminal nerve or the olfactory system [110]. Some theories suggest that oral microbiota could migrate to the gastrointestinal tract and impact the gut–brain axis [111,112]. However, research on the communication between oral microbiota and the brain remains limited. Additionally, the easy accessibility of the oral cavity positions it as a potential diagnostic tool for detecting microbial communities and predicting oral and systemic diseases, including neurological disorders.

Oral dysbiosis in ASD populations leads to alterations in the metabolome, resulting in increased metabolites strongly associated with ASD pathogenesis, such as acetate, propionate, and indoles, while butyrate levels decrease [112]. These changes confirm the central role of tryptophan metabolism. Literature reviews highlight a close connection between oral dysbiosis and the characteristic symptoms of ASD. Reconstructing oral and gut microbial ecosystems through probiotics could significantly reduce ASD symptom severity [111]. A growing body of evidence supports a direct correlation between ASD and the microbiome [113]. Several studies, primarily in animal models, have shown significant differences in gut microbiome composition and diversity between autistic and neurotypical individuals [114]. These findings suggest that gut dysbiosis may be a key cofactor in ASD pathogenesis. Some studies suggest that ASD can induce lifestyle changes, such as specific dietary preferences, which in turn promote gut dysbiosis [115]. This imbalance in gut microbial communities, known as dysbiosis, significantly impacts the gut–brain axis due to the close connection between the gut microbiome and the endocrine, immune, and enteric nervous systems [116]. Besides the gut microbiota, the oral cavity hosts the second most abundant microbial ecosystem in humans, playing a significant role in both health and the development of various diseases [117].

While establishing a direct causal link between oral dysbiosis and systemic disorders is complex, analyzing oral microbiota could serve as a strategic tool to better understand various human diseases’ origins and clinical progression [118]. Like the gut–brain axis, the oral microbiome may participate in bidirectional communication with the central nervous system [119]. It has been suggested that oral microbiota may influence or exacerbate the development of neuropsychiatric disorders [120]. The migration of specific bacteria from the mouth into the bloodstream may trigger harmful mechanisms, including systemic activation of pro-inflammatory cytokines, causing neuroinflammation, colonization of pathogens in various organs and tissues, and the dissemination of toxic microbial metabolites [121].

Activating these mechanisms can have severe consequences, including compromised blood–brain barrier (BBB) integrity, thus allowing toxic metabolites to enter the central nervous system (CNS) and interfering with the metabolic pathways that regulate synaptic transmission. These processes contribute to neuronal loss and synaptic deficits, leading to cognitive and behavioral deterioration. Oral dysbiosis in ASD may be reflected in altered metabolic profiles, with overproduction of metabolites closely linked to the disorder’s pathogenesis [122]. The increase in acetate and propionate, along with a reduction in butyrate and microbial indole synthesis, underscores the importance of biochemical processes in ASD, with particular attention to tryptophan metabolism [123]. It remains to be determined whether metabolic changes stem from oral dysbiosis in children with ASD. Oral microbiota may exhibit similar pathogenic mechanisms across conditions, but its composition varies by specific diseases. Furthermore, mental health disorders and neuropsychiatric conditions can cause behavioral changes, such as poor oral hygiene, smoking, and excessive alcohol consumption, which may contribute to periodontal disease and alter oral microbiota.

The potential role of the microbiome in ASD is significantly supported by various animal studies, which have shown that dysbiosis can alter social behaviors and that restoring gut microbes can improve symptoms [124,125]. Similar results have been observed in humans with ASD. For instance, antibiotic treatments with vancomycin have temporarily reduced some behavioral symptoms in ASD patients [126,127]. Additionally, a recent fecal microbiota transfer therapy study in 18 children with ASD found increased bacterial diversity and parent-reported improvements in gastrointestinal and behavioral symptoms [128]. These effects were observed up to eight weeks after the intervention, suggesting the potential long-term benefits of such therapies.

The oropharynx, the sole entry point to the gastrointestinal tract, is a site of interest in ASD pathology [13]. Children with ASD often exhibit motor impairments, such as those related to speech [129], sensory issues, and food structure preferences [51]. Epitranscriptomic variations in the saliva of children with ASD have been previously analyzed [130], leading to the hypothesis that oral microbiomes might also be altered in children with ASD.

The study by Evenepoel et al. [15] explored the human oral microbiome using advanced shotgun metatranscriptomic data from the oropharynx of 180 children with ASD, 106 typically developing (TD) children, and 60 children with non-autistic developmental delays (DD). This study represents the most extensive investigation of the oral microbiome in children with ASD and the first to utilize oropharyngeal samples. The oropharynx is a crucial point of interaction between the host and the environment. Sensory and motor innervation of the oropharynx is provided by five cranial nerves (V, VII, IX, X, and XII), which establish significant connections between the oropharynx and the CNS. These nerves provide a plausible communication route for the gut–brain axis, which also exerts significant influences via cranial nerve X [131].

Unsurprisingly, microtranscriptomic profiles exhibit distinct variations in children with ASD suffering from gastrointestinal disorders. Many of these “alterations” are also linked to specific features of ASD. For example, *Micrococcus luteus* levels are reduced in children with gastrointestinal disorders and those with gastrointestinal and adaptive communication difficulties [132]. Similarly, *Riemerella anatipestifer* and *Actinobacteria* levels show correlations with restricted/repetitive behaviors and social effects, respectively, and are “altered” in children with gastrointestinal disorders [132]. These trends are particularly significant, considering that ASD phenotypes not associated with gastrointestinal issues (e.g., ADHD) do not exhibit microbiome profile differences at the phylum or species levels.

In light of recent studies highlighting the role of genetic factors in ASD [8], it is unlikely that microbiome changes are the sole determinant of autistic behavior. Nonetheless, microbiome alterations have been associated with atypical social, communicative, and repetitive behaviors in animal models [124,125]. A potential mechanism of this association may involve metabolic disturbances [133]. The study demonstrated that microbial RNA profiles in children with ASD (compared to children with developmental delays and typically developing peers) exhibit significant alterations in oropharyngeal metabolic pathways [133].

Furthermore, a significant increase in microbial RNAs involved in lysine degradation was identified in the oropharynx of children with ASD. Lysine is a ketogenic amino acid whose degradation produces glutamate, a neurotransmitter essential for learning and memory. Elevated glutamate levels have been observed in the plasma [134,135] and CNS of ASD patients [14,136]. Evidence indicates increased “energy metabolism” and “carbon metabolism” in the oral microbiota of children with ASD compared to TD and DD peers. Energy metabolism encompasses several subcategories, including oxidative phosphorylation, photosynthesis, carbon fixation, methane metabolism, nitrogen metabolism, and sulfur metabolism.

Further analysis clearly indicates that the increase in “energy metabolism” in children with ASD is primarily due to heightened expression of bacterial transcripts associated with oxidative phosphorylation (1.6-fold) and methane metabolism (1.2-fold) [137,138]. Notably, oxidative phosphorylation, which involves the QCRC2 pathway (associated with CNS diseases such as Alzheimer’s and Parkinson’s), strongly correlates with cyanobacterial activity. The cyanobacterial activity was significantly higher in ASD participants than in neurotypical controls [139,140].

Another mechanism by which host–microbe interactions may influence altered social behaviors involves toxicological effects [109]. For instance, changes in oral cyanobacteria in children with ASD were observed at both the phylum and species levels. The study also demonstrated that cyanobacteria levels could distinguish autistic children from their neurotypical peers (TD). Cyanobacteria, environmental pathogens found in contaminated water, produce cyanotoxins that can cause severe illnesses such as gastrointestinal disorders, hay fever, and itching. Additionally, the neurotoxin β-N-methylamino-L-alanine, produced by cyanobacteria, has been suggested as a contributing factor to neurodegenerative conditions like Parkinson’s and Alzheimer’s. Son et al. [141] previously reported alterations in cyanobacterial levels in the fecal microbiome of children with ASD compared to neurotypical siblings.

#### 3.4.2. Microbiome as a Biomarker

Assuming the hypothesized correlation between alterations in gut microbiota and ADHD/ASD, research has examined the composition of gut microbiota in children and adolescents with and without these conditions, assessing the systemic effects of the involved bacteria. While these disorders have precise genetic components [9], heredity alone does not fully account for their development. It is proposed that ADHD and ASD may arise from complex interactions between genetic and environmental factors [142].

Both ADHD and ASD are frequently associated with gastrointestinal symptoms such as constipation and abdominal pain [143,144], indicating that gastrointestinal dysfunction may contribute to these disorders [145,146]. Additionally, children with ASD exhibit a distinct gut microbiota compared to unaffected controls. For both ADHD and ASD, low-grade systemic inflammation has been observed, likely connected to the translocation of bacterial products across the gastrointestinal epithelial barrier [17]. Increased intestinal permeability has been documented in both ADHD and ASD, suggesting that dysfunction of the intestinal barrier might play a role in the pathogenesis of these disorders [147].

It remains unclear if changes in bacterial composition indicate a pathological alteration of gut microbiota or reflect impaired gastrointestinal function in children with ADHD or ASD. However, a higher relative abundance of *Eggerthella* has been previously associated with developmental delays in children [148].

The GEMMA (Genomic, Environmental, Microbiome, and Metabolomic Assessment) project is a multicentered, prospective, open-label, uncontrolled study with both observational and interventional arms [149]. It is conducted through an international collaboration involving research institutions in the European Union and the United States. This project is coordinated by the European Biomedical Research Institute of Salerno (EBRIS), located in Campania, Italy.

The primary aim of GEMMA is to study the interaction among genomic, environmental, microbiome, and metabolomic factors, which, in synergy with the immune system, may influence the development of ASD. The study adopts a longitudinal approach, analyzing how these variables evolve over time and seeking to identify the underlying mechanisms that may contribute to ASD onset. This project represents an innovative approach aimed at understanding how these factors interact, hoping to identify new prevention and treatment strategies for ASD. It includes observational clinical studies as well as experimental research. On one side, humanized mouse models (mice receiving fecal transplants from ASD individuals) and in vitro colon models are utilized. On the other side, children at risk of developing ASD, along with their siblings already diagnosed, are recruited.

This approach will help identify specific microbiome signatures and metabolic pathways involved in ASD progression and severity, as well as potential treatment responses. The study will also account for environmental factors such as delivery type, neonatal feeding methods, and the introduction of solid foods and allergens into the diet, which may be correlated with ASD development.

A deeper understanding of the interactions between gut microbiota and host genomics is crucial to predict how these interactions influence the metabolic pathways responsible for clinical and functional outcomes. GEMMA aims to revolutionize the approach to ASD primary prevention by manipulating gut microbiota, shifting the focus toward a new treatment paradigm. Identifying specific metagenomic and metabolomic phenotypes linked to ASD could facilitate the development of new diagnostic tools based on biomarkers and targeted therapeutic strategies. Furthermore, the GEMMA biorepository will support future epigenetic studies and validation of these biomarkers [150].

### 3.5. Analysis of Existing Therapeutic Approaches to Manage Oral Self-Harm in Patients with ASD

Effective management of oral self-harm in patients with ASD requires a multidisciplinary approach, combining behavioral strategies, pharmacological treatments, and microbiota-targeted therapies. Recent research highlights the potential role of microbial interventions, such as probiotics (e.g., *Lactobacillus reuteri*, *Bifidobacterium longum*, and *Lactobacillus plantarum*), in modulating the gut microbiota and influencing behavioral responses [128,151]. A double-blind, randomized, placebo-controlled trial demonstrated that precision microbial therapies could improve social behavior but do not significantly alter the core symptoms of ASD [152]. These findings suggest that microbiota-based treatments could be integrated with conventional therapeutic strategies to enhance patient outcomes. Understanding the complex interplay between neurodevelopment, oral health, and microbiota is essential for developing targeted interventions to reduce self-injurious behavior (SIB) and improve oral health management in individuals with ASD [128,151].

After diagnosis, oral therapy emphasizes symptomatic treatment to minimize tissue damage caused by self-injurious behavior (SIB). However, addressing the underlying impulses behind the behavior is essential for successful treatment, requiring pharmacological and behavioral therapies. Cognitive-behavioral approaches are more effective than applied behavioral analysis in these cases.

Symptomatic treatments to protect oral tissues include using a mouthguard, although patient compliance is necessary. In some instances, self-injurious behaviors are so severe that they pose a health risk to the individual. Therefore, it is often crucial to complement therapeutic interventions with pharmacological treatment. Risperidone, an antipsychotic medication, is commonly used in such cases. It seems effective in the short-term treatment of tantrums, aggression, and SIB in children with autism spectrum disorder (ASD), and improvements in stereotypic behavior and hyperactivity have also been observed. However, it should be used cautiously due to the high risk of adverse effects and uncertainty about long-term outcomes. Current guidelines suggest that interventions aimed at reducing aggression should precede medication use. Many patients with ASD who exhibit agitation and aggressive behaviors may not respond to pharmacotherapy [153].

Containment techniques and environmental modifications can temporarily prevent physical injury to protect an individual’s physical integrity. However, these techniques are generally considered a last resort, as protocols aim to prevent and eliminate such behaviors.

Cognitive-behavioral therapy (CBT) is one of the most common therapeutic interventions, especially in cases where self-injury is used to obtain or avoid specific outcomes. The most frequently employed methods within this approach involve replacing dysfunctional behaviors with more appropriate and incompatible ones, scheduling self-stimulatory behaviors within designated times and spaces, and thus permitting certain behaviors only during predetermined periods and locations. It is crucial to anticipate stressful situations or to prevent and minimize stimuli that may trigger these dysfunctional behaviors. A communication system that allows the individual to express their needs is essential, as this can reduce tension and frustration, thereby decreasing self-injurious behaviors. However, it is noteworthy that such behaviors may often be beyond the individual’s control.

Occupational therapy based on sensory integration exposes children to a range of stimuli, aiming to integrate them in a fully controlled environment created by the therapist. The therapist evaluates which stimuli generate stress and which have a calming effect. Schaaf has widely studied this approach, identifying significant benefits in play and social interactions. Both areas are closely linked to dysfunctional self-stimulation behaviors, which tend to isolate the individual [154].

Ayres’ sensory integration approach in occupational therapy is a clinical and rehabilitative method rooted in the neuroscientific neuroplasticity principle [65]. It suggests that nerve cells adapt and modify their functioning to ensure survival. Techniques focused on habituation and awareness have shown positive results in reducing self-stimulatory or self-injurious behaviors without side effects. Therapy rooms are custom designed for patients, ensuring comfort and a sense of control. Furthermore, engaging in motivational and recreational activities stimulates the limbic system, leading to effective learning, the development of self-regulation skills, and enhanced communication abilities and executive functions.

When pharmacotherapy proves insufficient, maintenance electroconvulsive therapy (M-ECT) may be considered. While not part of routine treatment protocols, it is rarely used in any age group. Studies indicate that catatonia is often associated with autism spectrum disorder (ASD), and ECT has been shown to be effective in addressing this issue. Additionally, self-injury can be viewed as a stereotypical symptom of catatonia [155].

The primary goal of therapy is to calm patients rather than sedate them. Four different approaches are utilized in treating aggressive patients: environmental organization, calming techniques, somatic restraint or isolation, and pharmacological therapy. A study involving various patients diagnosed with ASD showed a significant reduction in self-injurious behaviors following ECT; however, many patients experienced relapses after treatment cessation. It can be concluded that treating catatonic comorbidities in ASD with benzodiazepines and/or ECT is adequate. However, there is no consensus on the use of ECT in children and adolescents.

According to the literature, early use of ECT can be beneficial in managing the disorder, particularly in cases of catatonia and, more rarely, in cases of agitation. Possible side effects of ECT include cardiometabolic and endocrine effects (such as weight gain and hyperprolactinemia) and extrapyramidal symptoms. Patients with severe self-injurious behaviors may require long-term maintenance ECT (M-ECT), typically involving monthly treatment sessions. M-ECT can be considered a viable treatment option for ASD cases with comorbid aggression and catatonia.

#### 3.5.1. Dermatillomania: Targeted Intervention Strategies

No intervention may be necessary in subclinical cases, while treatment is recommended when diagnostic criteria are met.

There are limited data regarding treatment options. The management of dermatillomania is currently based on a comprehensive psychiatric evaluation, behavioral therapy, and medications.

Non-pharmacological treatments include the following:

- Cognitive-behavioral therapy (CBT): This approach involves psychoeducation, cognitive restructuring, and relapse prevention through self-esteem enhancement, along with clearly defined strategies to prevent or manage potential relapses [156].
- Habit reversal training (HRT): Previously used to treat a variety of repetitive behavior problems, such as cheek biting, oral-digital habits, and trichotillomania (TTM19) [157]. It includes the following:
  ○ Awareness training (self-monitoring): Teaching the patient to recognize skin-picking triggers and behavior.
  ○ Competing response training: The patient learns to replace skin-picking with an incompatible action, such as clenching their fist.
  ○ Decoupling (DC): The patient is trained to “unlearn” skin-picking by replacing it with a harmless behavior that mimics the central movements of the problematic behavior, such as bringing the hand close to the face without picking and then redirecting it to a different location, such as the ear, where picking does not occur.

From a pharmacological perspective, the following have been tested:

- Selective serotonin reuptake inhibitors (SSRIs) have not demonstrated significant changes [158].
- Lamotrigine: Shown to yield positive results [157].
- Glutamatergic agents, such as N-acetylcysteine (NAC) and riluzole, have shown notable improvements [158].
- Opioid antagonists: Such as naltrexone [159].

Additionally, alternative treatments have been identified, including yoga, aerobic exercise, acupuncture, and hypnosis, either as monotherapy or as an adjunct to psychotherapy and/or pharmacotherapy.

#### 3.5.2. Probiotics

Probiotics, according to the Food and Agriculture Organization (FAO) of the United Nations and the World Health Organization (WHO), are defined as “live microorganisms which, when administered in adequate amounts, confer a health benefit on the host.” They enhance the homeostasis of the internal microbiota to maintain intestinal health [160]. Probiotics reduce the number of harmful bacteria, which struggle to survive in acidic environments, while increasing beneficial bacteria that thrive under these conditions, thereby restoring the balance of the intestinal microbiota.

Some studies suggest combining probiotics with prebiotics and recommend the use of symbiotics.

The term “prebiotics” is relatively recent and refers to “non-digestible food ingredients that benefit the host by selectively stimulating the growth and/or activity of specific bacteria in the colon, thereby improving host health” [161]. In 2017, experts revised this definition, describing prebiotics as “a substrate selectively utilized by host microorganisms to confer a health benefit.” This updated definition broadens the scope of prebiotics to include potentially other substances, applications in body areas beyond the gastrointestinal tract, and categories outside food [162].

On the other hand, symbiotics refer to the combined use of probiotics and prebiotics to improve human or animal health [163]. In symbiotic products, prebiotics provide a specific substrate that supports the growth of probiotic bacteria [164,165,166]. Numerous studies suggest that consuming symbiotics can positively impact health and nutritional status by supporting healthy gut microbiota and improving overall well-being.

According to current scientific literature, alterations in gut flora may be closely linked to the development of ASD. Some studies have aimed to determine whether probiotic supplementation could have a protective effect on individuals with ASD. In recent years, more studies have confirmed that probiotic supplementation may be effective for children with ASD. According to Sherwin et al., probiotics can influence the central nervous system (CNS) and modulate the secretion of hormones such as oxytocin and cortisol, produced by the pituitary and adrenal glands, respectively [167]. This process occurs through the gut–brain axis, a bidirectional communication system between the gastrointestinal tract and the CNS that involves the immune, endocrine, and autonomic nervous systems.

A meta-analysis assessed the effectiveness of probiotic supplementation in children with ASD. The studies included were conducted in countries like Italy, the United States, Taiwan, and several cities in China, involving subjects aged 3 to 14. The objective was to evaluate whether probiotic supplementation could improve social skills, developmental quotient, and overall clinical impression in children with ASD. The results showed that probiotics did not significantly improve social skills. Furthermore, no significant increases were observed in developmental quotient or global clinical impression. Similar results were reported in earlier studies, such as that by Song et al., which found that neither probiotics nor prebiotics significantly improved the severity of ASD symptoms, gastrointestinal issues, or comorbid psychopathology [168]. However, probiotics effectively reduced gastrointestinal symptoms, suggesting their potential as a therapeutic strategy for neurodevelopmental disorders by lowering levels of 4-ethylphenyl sulphate (4EPS), identified as a potential biomarker of human ASD [169].

Various factors have been analyzed to explain why probiotics may not effectively improve ASD symptoms. Firstly, the pathophysiological mechanism of ASD is highly complex and multifactorial, so probiotic supplementation alone may not be sufficient to achieve significant improvements. Secondly, results may vary depending on the type of probiotics used: single-strain or multi-strain probiotics, with varying efficacy. Additionally, many studies fail to specify the dosage or manufacturers of the probiotics, introducing variability in the findings. Lastly, gastrointestinal conditions like constipation and diarrhea can influence probiotic absorption, reducing their effectiveness.

Children with ASD have been found to exhibit significant imbalances in gut microbiota, characterized by a reduced Bacteroidetes-to-Firmicutes ratio and increased levels of Lactobacilli. Probiotic supplementation has shown improvements in behaviors, particularly reducing destructive tendencies and anxiety, while promoting better social interaction and cognitive function. These findings suggest that balancing gut microbiota could positively impact behavioral symptoms associated with ASD [169]. Therefore, probiotics and ASD may be closely linked. A study by Zeng P et al. [151] reported significant improvements in gastrointestinal function, with a reduction in the total gastrointestinal severity index in the probiotic-treated group. Probiotics contain microorganisms similar to the beneficial bacteria naturally present in the human gut, and the gut microbiome can communicate with the brain through microbiota-derived signaling molecules, immune mediators, gut hormones, and vagal and spinal afferent neurons, potentially influencing brain activity and ASD symptoms [170,171].

Probiotics have improved results in various ASD assessment scales, though the differences were not statistically significant. A study by Ning Sun et al. highlighted that compound probiotics could support physical growth by improving intestinal development [172]. These findings suggest that while probiotics may have a limited direct effect on ASD behavioral symptoms, they can contribute positively to children’s general health and physical development through enhanced intestinal functionality. Another study suggested that probiotics might reduce body mass index and hip circumference, effects that, if not carefully monitored, could hinder growth and development in children [173].

The role of probiotics in children with ASD remains controversial and requires further research. Systematic reviews and meta-analyses indicate that while probiotics may alleviate gastrointestinal symptoms, no statistically significant differences are observed in behavioral, social, physical, or mental development, nor in overall improvement in children with ASD. These studies suggest that despite the potential of probiotics to modulate the gut–brain axis and influence ASD symptoms, current evidence does not support their effectiveness in improving social and cognitive aspects in children with ASD. These results highlight the need for further studies to clarify the potential role of probiotics or other interventions in the treatment of ASD.

Meta-analyses indicate that probiotic supplementation reduces gastrointestinal symptoms in children with ASD but shows limited efficacy in improving social or cognitive functions. Probiotics may positively influence anxiety, destructive behaviors, and social aptitude by rebalancing the microbiota. Variability in results may stem from differences in probiotic strains, dosages, and individual gastrointestinal condition [174].

##### Challenges

The multifactorial nature of ASD complicates treatment, and probiotics alone may be insufficient to yield significant improvements. Studies underscore the need for further research to clarify their therapeutic potential. In conclusion, while probiotics are promising to alleviate gastrointestinal issues and indirectly influence behavior, their direct impact on core ASD symptoms remains unproven [175]. Future studies should focus on standardized protocols, long-term effects, and synergistic treatments to better assess their role in ASD management.

## 4. Discussion and Conclusions

**Figure 6:**
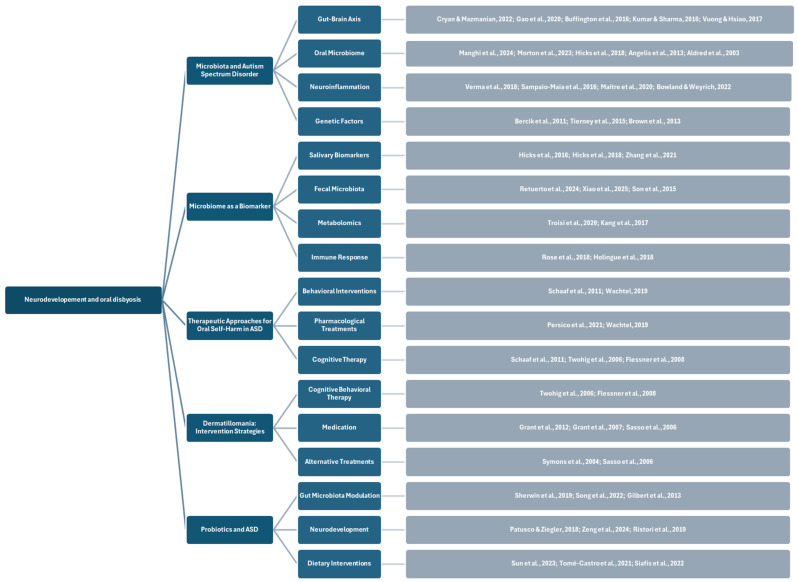
Conceptual map of microbiota, ASD, hypotheses, and therapeutic interventions. This conceptual map illustrates the relationships between microbiota, ASD, and different therapeutic approaches. The primary categories include microbiota and ASD [109,112,113,116,117,118,119,120,123,124,125,129,131,132,133,134,136], microbiome as a biomarker [128,130,132,139,140,141,144,146,148,149], therapeutic approaches for oral self-harm in ASD [153,154,155,156,157], intervention strategies in dermatillomania [156,157,158,159,160,161] and probiotics and ASD [151,167,168,170,171,172,173,174,175]. Each category is further divided into specific subtopics (branches).

**Figure 7:**
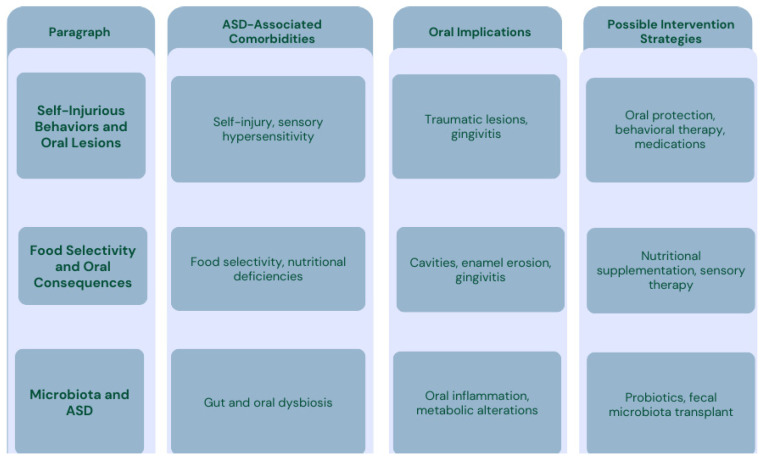
Overview table of the results.

The results of this study have brought to light the complex, interrelated relationship between ASD, oral health, and microbiota and related potential therapeutic strategies (Figure 6 and Figure 7).

Individuals with ASD are at an increased risk of developing unique oral health issues related to their heightened sensory sensitivities, selective dietary habits, self-injurious behaviors, and difficulties with routine oral hygiene practices. Such actions might increase the chance not only of dental diseases such as gingivitis, enamel hypoplasia, caries, and traumatic lesions but also of the emergence of systemic disturbances based on oral microbial imbalance and dysbiosis of gut microbiota. Thus, there was a considerable association among oral-gut microbiota, neurodevelopment, and behavioral problems in ASD by routes such as gut–brain or oral–brain pathways, stating the presence of a bidirectional relationship between the symptoms [176].

This study also sheds light on the highly prevalent nutritional deficiency among ASDs related to food selectivity and feeding difficulties. Deficiencies of essential vitamins A, C, and D, along with an imbalance in micronutrients, worsen oral health and might further deteriorate systemic health [83]. For example, low consumption of fiber and specific food groups required for gut microbiota homeostasis promotes intestinal dysbiosis and downstream neuroinflammation and immune responses. These findings support the importance of early and targeted interventions focused on these nutritional gaps, remarkably increasing dietary variety and managing sensory sensitivities to foster healthier eating behaviors.

Different therapeutic approaches targeted at the microbiota, such as probiotics and fecal microbiota transfer, show promise in rebalancing microbial ecosystems and sometimes even alleviation of symptoms of ASD [128,170]. However, their efficacy in improving core behavioral and cognitive symptoms remains to be fully established, possibly reflecting the multi-factorial etiology of ASD. A recent pilot double-blind, randomized, placebo-controlled trial demonstrated that precision microbial interventions could improve social behaviour but do not significantly alter ASD severity, underscoring the need for further investigation into their therapeutic potential [152].

Aside from the microbiota’s impact on serotonin production, short-chain fatty acid metabolism, and neuroinflammatory pathways, the potential intervention points are complex and require far more research. In addition, sensory integration therapies and behavioral interventions may help improve oral health and reduce maladaptive behaviors [12].

Combining such approaches with microbiota-focused therapies could lead to a more holistic, integrative approach to care. The GEMMA project is one such model for the value of this research approach, looking at the interplay of genetic, environmental, and microbiome factors in ASD. By pointing out specific microbial and metabolic biomarkers related to ASD, GEMMA opens new avenues in early diagnosis, targeted treatment, and personalized care. Such an integrative approach points to the need to address ASD’s complexity with a collaborative interdisciplinary approach across neurology, dentistry, gastroenterology, nutrition, behavioral science, and psychology so that interventions address everyone’s needs [177]. It could result in deepening our understanding of the microbiota–systemic health relationship in ASD and support the creation of innovative care models to improve the quality of life for individuals with ASD and their families.

Future studies should focus on refining these therapies, testing their long-term efficacy, and integrating them with other interventions such as sensory integration and cognitive-behavioral therapies. Moreover, given the clinical variability of ASD and other neurodevelopmental disorders (NDDs), further investigation is needed to develop more standardized diagnostic evaluations that account for phenotypic differences, particularly between males and females, and to address their specific clinical needs. Since nutritional deficiencies and feeding problems are key aspects of ASD, early and personalized dietary interventions are essential to managing comorbidities and improving overall health and well-being.

These efforts will deepen our understanding of the microbiota-systemic health relationship in ASD and support the creation of innovative care models to improve the quality of life for individuals with ASD and their families.

### Study Limitations

Although this study provides valuable insights into the relationship between ASD, oral health, and microbiota, several limitations should be acknowledged, particularly regarding the methodology.

Firstly, although our literature review employed a systematic search strategy across multiple databases, the lack of a standardized protocol for selecting and analyzing studies may impact the replicability of our findings. Future research should adopt more rigorous methodologies, such as PRISMA guidelines, to ensure transparency and reproducibility.

Secondly, our study relies on existing literature, which varies in methodological quality. Many studies in our review differ in sample sizes, study designs, and diagnostic criteria for ASD, leading to potential inconsistencies in the reported outcomes. A meta-analysis with stricter inclusion criteria would strengthen the reliability of these associations.

Additionally, the lack of longitudinal studies assessing the long-term effects of microbiota-targeted interventions, such as probiotics or dietary changes, limits our ability to determine their sustained impact on ASD-related symptoms. Future studies should implement controlled trials with well-defined protocols to validate these therapeutic approaches.

Finally, while we discuss the potential mechanisms underlying the gut–brain and oral–brain axes in ASD, the current body of research is still developing. More experimental studies are needed to confirm causality rather than correlation in microbial imbalances and their neurodevelopmental effects. Despite these limitations, this study underscores the importance of integrating microbiota research into ASD management and highlights promising avenues for future investigation. Addressing these methodological challenges will be crucial for advancing our understanding of the complex interactions between ASD, oral health, and the microbiome.

## Data Availability

No new data were created or analyzed in this study.

## Abbreviations

The following abbreviations are used in this manuscript:

ASD	autism spectrum disorder
SIB	self-injurious behavior
PDD-NOS	pervasive developmental disorder not otherwise specified
GEMMA	Genomic, Environmental, Microbiome, and Metabolomic Assessment
ADHD	attention-deficit/hyperactivity disorder
NDD	neurodevelopmental disorders
